# An Effective Method to Model and Simulate the Behavior of Inductive Angle Encoders

**DOI:** 10.3390/s22207804

**Published:** 2022-10-14

**Authors:** Robert Alexander Dauth, Gerald Gerlach, Sina Fella

**Affiliations:** 1 Robert Bosch GmbH, Robert-Bosch-Allee 1, 74232 Abstatt, Germany; 2 Faculty of Electrical and Computer Engineering, Technische Universität Dresden, Helmholtzstraße 10, 01069 Dresden, Germany

**Keywords:** angle encoders, rotary encoders, inductive sensors, mutual coupling, finite-element simulation, design of experiments, optimal designs, linear regression

## Abstract

Many angle or position sensors rank among the inductive encoders, although their sensing principle is different. The sensor design investigated in this paper is based on coupled coils, whereas the information about the position angle is modulated on the induced voltage, measured at the receiving coils. Unfortunately, no closed solution for most of the physical quantities exists, since this principle is based on eddy currents, which are rather complex to calculate for the given geometry. Consequently, the common way is to calculate the sensor quantities by a 3D finite-element (FE) simulation. However, this leads in most cases to a high time and computational effort. To overcome the limitations with respect to computational resources, a novel method is presented to reduce simulation effort and calculate regression models, which can even replace simulations. In the following investigations, D-optimal designs are used—a subdomain in the field of statistical design of experiments—and combined with a numerical implementation of Faraday’s law, in order to calculate the induced voltages afterwards from simulated magnetic field data. With this method, the sensor signals can be calculated for multiple angle positions from one simulated position by shifting the integration boundaries. Hence, simulation time is significantly reduced for a full period. The regression models obtained by this method, can predict the Tx-coil inductance, induced Rx-voltage amplitude and angular error in dependency of geometric design parameters.

## 1. Introduction

This work contributes to inductive angle encoders. Many angle encoders—independent of their operating principle—share one typical characteristic: The angle is not measured directly, but rather encoded in two orthogonal signals—commonly known as sine and cosine channels in quadrature—that form a vector in the complex plane. The actual angle is then calculated with the (unwrapped) arctangent function, essentially converting Cartesian coordinates to a polar angle.

**Figure 1:**
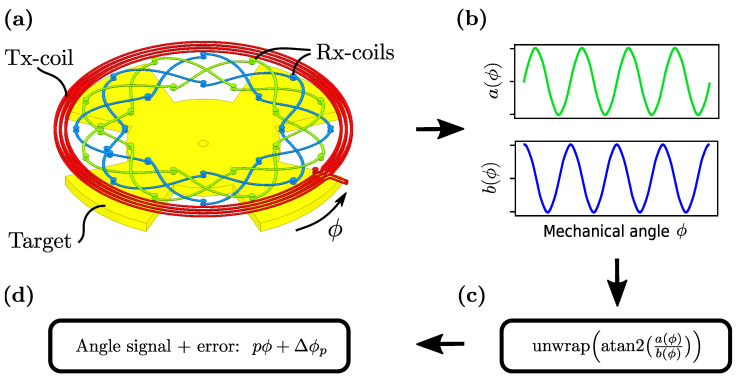
Overview of the sensor coil system (**a**)—including a target—and the signal flow from encoder signals (**b**) to the calculated angle and error (**d**) by calculation of the *unwrapped* arctangent function (**c**). Note that the signals *a* and *b* are already demodulated. The figure does not consider the signal-processing circuitry.

The following investigations focus on a printed circuit board-based (PCB) sensor design which contains a planar coil system—comprising a transmitter coil (Tx-coil) and at least two receiver coils (Rx-coils)—a rotating coupling element (target) made out of conductive material and a signal-processing circuitry for measuring, demodulation and amplification of the coil-signals (Figure 1). For this sensor design the position angle can be derived from the induced voltage signals on the Rx-coils. The Tx-coil is arranged in an oscillator circuit in order to generate a time-varying magnetic field. Due to the concentric arrangement of the coils on the PCB, this magnetic field infuses the Rx-coils together with the target, which is placed directly above the coils in a certain distance (air gap).

In absence of a target the coupling between the Tx- and Rx-coils is zero and thus also the induced voltage signals, since the geometry of the Rx-coils leads to a differential signal. If a target is present, the magnetic field of the Tx-coil induces eddy currents, that—pursuant to Lenz’s law—create an opposing field. Accordingly, the magnetic field below the target almost cancels out, but yields an inhomogeneous field distribution in the angular direction above the Rx-coils. A rotation of the target therefore leads to an amplitude-modulated voltage signal on the Rx-coils, that oscillates with the carrier frequency of the Tx-oscillator. Note that within this paper dynamical effects—caused by high rotational speeds—are neglected (quasi-static).

In order to demodulate and amplify the induced voltage signals on the Rx-coils, an output circuitry is needed. Commonly, an application-specific integrated circuit (ASIC) is chosen for that use, since it includes most of the necessary circuitry in one electronic part. Based on the application and the utilized signal-processing circuitry or ASIC, the coil system has to fulfill several requirements. For example, the amplitude of the induced voltage signals needs to be in a specific range depending on the amplifier stage. Unfortunately, no closed analytic solution exists for the coil system quantities. This is mostly related to the eddy current distribution, which is rather complex to calculate for the given geometry. Analytical solutions exist mainly for simple geometries as shown by Nagel [1]. The common way to calculate the sensor quantities numerically is a finite-element (FE) simulation. Unfortunately, most simulation tools are commercial software and require high computational resources and time-effort.

Throughout the literature there are many publications about the application of statistical methods—especially considering optimal designs—on modeling of physical quantities of various sensor types. For example, Gianchandani and Crary presented a similar approach for the modeling of a microaccelerometer, by creating also optimal designs [2]. However, in terms of the inductive position sensing principle based on coupled coils, this method was not applied yet. Regarding this sensor principle, Passarotto et al. showed a time-efficient simulation approach which uses a surface integral method [3]. Hoxha et al. applied this method in combination with a *non-linear least-squares solver*, in order to optimize the sensor accuracy by changing the Rx-coil geometry [4]. However, the current literature does not provide an in-depth overview about the sensor behavior in dependency of geometric parameters. State of the art is the estimation of various sensor quantities by numerical calculation in form of a FE simulation. An alternative to FE calculations is not presented for this sensor design.

**Figure 2:**
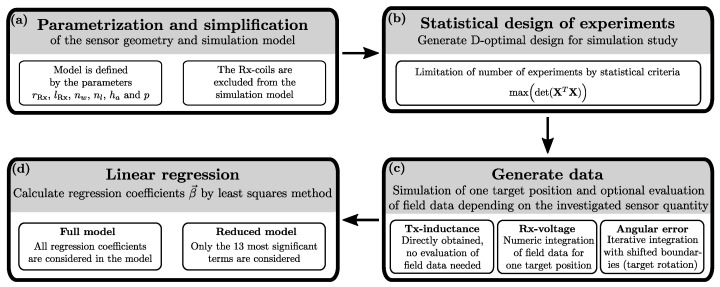
Modeling approach starting with a parametrization and simplification of the sensor geometry (**a**), followed by application of statistical methods (**b**), data generation (**c**) and calculation of regression models (**d**). The geometric parameters of the sensor model are the intermediate Rx-coil radius $r_{\mathrm{Rx}}$, the Rx peak-to-peak distance $l_{\mathrm{Rx}}$, the number of Tx-windings per layer $n_{w}$, the number of Tx-layers $n_{l}$, the air gap between coils and target $h_{a}$ and the periodicity of the sensor *p*. For the statistical methods the design matrix X, the response variable $\vec{y}$ and the regression coefficients $\vec{\beta }$ must be introduced.

The novel method presented in this paper combines model simplification and numerical calculations with statistical methods, aiming at the reduction of simulation effort. Simulation software will still be used for data generation, but the computational resources are reduced to a minimum (Figure 2). At the end, regression models are calculated which are able to predict sensor quantities—depending on various parameters—without any further simulation.

## 2. Modeling and Simulation Method

The characteristics of inductive sensors which are based on coupled coils are mostly dependent on the geometry and the arrangement of the individual sensor components. Hence, the following studies focus on the influence of geometric design parameters. A generalized simulation model will be used for the investigations, which is dependent on the intermediate Rx-coil radius $r_{\mathrm{Rx}}$, the Rx peak-to-peak distance $l_{\mathrm{Rx}}$, the number of Tx-windings per layer $n_{w}$, the number of Tx-layers $n_{l}$, the air gap between coils and target $h_{a}$ and the periodicity of the sensor *p*. In order to reduce computational effort, the Rx-coils will be excluded from the simulation model. This is valid as the physical point of view will show, since the magnetic field distribution is—in good approximation—not influenced by the Rx-coils. The next step according to Figure 2 is to determine design parameter variations with a high informational output, considering a limited number of simulations. In the field of statistical design of experiments, *D-optimal designs* are used to minimize the covariance of estimated regression coefficients $\vec{\beta }$. If a design is not D-optimal, more experiments are needed to achieve the same prediction accuracy. *Design* means in this context a set of experiments—or in this case simulations—with different parameter variations. Once the set of simulations—given by a so-called *design matrix* X—is determined, the dataset of response values $\vec{y}$ can be generated for the following regression analysis. If the sensor quantity of interest is related to the induced voltage on the Rx-coils, a numeric integration of the simulated magnetic field data must follow. The most important benefit of this method is, that even for a full rotation only one target position needs to be simulated. The rotation can be represented by an iterative calculation with shifted integration boundaries. This is valid, due to the almost symmetric field distribution of the simplified coil system. After data are acquired by simulation and following numeric integration for the Rx-coil quantities, linear regression models can be fitted. For the calculation of the regression coefficients, the common least-squares method is applied.

### 2.1. Equivalent Circuit Model

**Figure 3:**
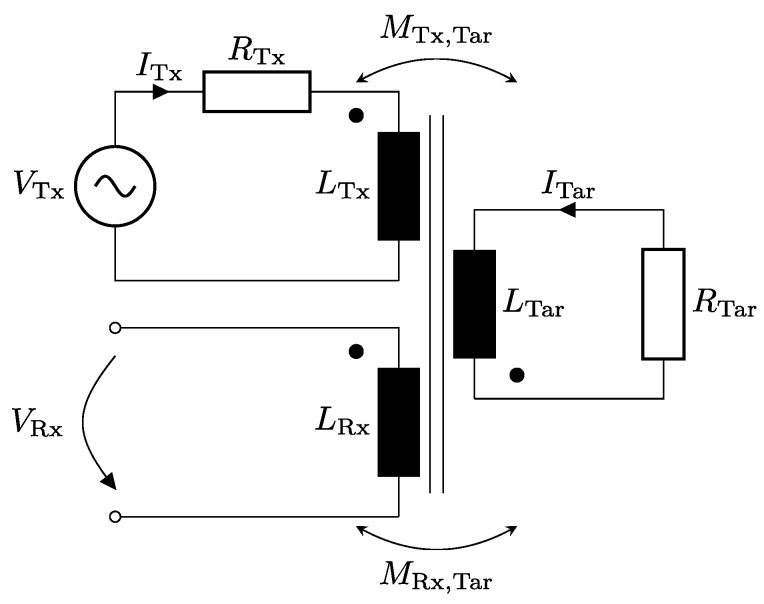
Equivalent circuit diagram of the coil system of the inductive coupled coil sensor. The oscillator driver for the Tx-coil is replaced by an AC-voltage source. This is a valid approximation which is also done in most simulation models. Note, that for simplicity just one Rx-coil circuit is drawn. The ohmic resistance of the Rx-coil is neglected since the amplifier stage input for the voltage measurement has a high impedance to avoid current flow.

As shown in Figure 3 the coil system including the target can be modeled as a transformer circuit. For simplification, the oscillator driver for the Tx-coil can be replaced by an AC-voltage source, which matches the definition in most simulation models. Furthermore, only one Rx-coil is considered, since in an ideal case the difference of both coils lies only in a phase-shift. All following considerations refer to an isolated environment without further conductive material—such as a housing—in close proximity to the sensor. At first, Ohm’s law is written down which results in a linear system for the whole network:

(1) $$\begin{array}{c} \begin{array}{cc} \left[\begin{array}{ccc} Z_{\mathrm{Tx}} & Z_{\mathrm{Tx}},\mathrm{Tar} & Z_{\mathrm{Tx}},\mathrm{Rx}\\ Z_{\mathrm{Tar}},\mathrm{Tx} & Z_{\mathrm{Tar}} & Z_{\mathrm{Tar}},\mathrm{Rx}\\ Z_{\mathrm{Rx}},\mathrm{Tx} & Z_{\mathrm{Rx}},\mathrm{Tar} & Z_{\mathrm{Rx}} \end{array}\right]\;\cdot \;\left[\begin{array}{c} I_{\mathrm{Tx}}\\ I_{\mathrm{Tar}}\\ I_{\mathrm{Rx}} \end{array}\right] & =\left[\begin{array}{c} U_{\mathrm{Tx}}\\ U_{\mathrm{Tar}}\\ U_{\mathrm{Rx}} \end{array}\right]\;,\\ \left[\begin{array}{ccc} R_{\mathrm{Tx}}+j\omega L_{\mathrm{Tx}} & -j\omega M_{\mathrm{Tx}},\mathrm{Tar} & 0\\ -j\omega M_{\mathrm{Tar}},\mathrm{Tx} & R_{\mathrm{Tar}}+j\omega L_{\mathrm{Tar}} & -j\omega M_{\mathrm{Tar}},\mathrm{Rx}\\ 0 & -j\omega M_{\mathrm{Rx}},\mathrm{Tar} & j\omega L_{\mathrm{Rx}} \end{array}\right]\;\cdot \;\left[\begin{array}{c} I_{\mathrm{Tx}}\\ I_{\mathrm{Tar}}\\ 0 \end{array}\right] & =\left[\begin{array}{c} U_{\mathrm{Tx}}\\ 0\\ U_{\mathrm{Rx}} \end{array}\right]\;. \end{array} \end{array}$$

The ohmic resistance of the Rx-coils is neglected, since it is a small value compared to the targeted very high resistance of the amplifier input. Therefore, the current through the Rx-coils is almost zero and hence neglectable. Note, that also proximity losses are not included inside the impedance matrix, since they are also considered to be comparatively small. Due to the reciprocity theorem follows for the mutual inductances [5,6,7,8]:

(2) $$\begin{array}{cc} M_{\mathrm{Tx},\mathrm{Tar}} & =M_{\mathrm{Tar},\mathrm{Tx}},\\ M_{\mathrm{Tar},\mathrm{Rx}} & =M_{\mathrm{Rx},\mathrm{Tar}}\;. \end{array}$$

According to the differential Rx-coil geometry it yields:

(3) $$M_{\mathrm{Tx},\mathrm{Rx}}=M_{\mathrm{Rx},\mathrm{Tx}}=0\;,$$

which means that there is no direct coupling between the Tx- and Rx-coils.

### 2.2. Tx-Coil Impedance

One important quantity of the coil system is the impedance of the Tx-coil in presence of the target. The imaginary part of this impedance determines—in combination with at least one capacitor—the frequency of the Tx-oscillator circuit. Since the mutual coupling between Tx- and Rx-coil is zero, the Rx-coil loop can be ignored in the network. Equation (1) is solved for the resulting impedance [9]:

(4) $$Z_{\mathrm{Tx},\mathrm{Tar}}=R_{\mathrm{Tx}}+\frac{\omega^{2}M_{\mathrm{Tx},\mathrm{Tar}}^{2}R_{\mathrm{Tar}}}{R_{\mathrm{Tar}}^{2}+\omega^{2}L_{\mathrm{Tar}}^{2}}+j\omega \left(L_{\mathrm{Tx}}-\frac{\omega^{2}M_{\mathrm{Tx},\mathrm{Tar}}^{2}L_{\mathrm{Tar}}}{R_{\mathrm{Tar}}^{2}+\omega^{2}L_{\mathrm{Tar}}^{2}}\right)\;.$$

Equation (4) shows, that even without considering proximity effects, the resulting ohmic resistance and inductance—which can be measured e.g., with an impedance analyzer—is mainly influenced by the self-inductance of the target and the mutual inductance between the Tx-coil and the target. Accordingly as an example, a change of the air gap between the target and the coil system would affect the ohmic resistance measured at the Tx-coil connections and also the oscillator frequency.

### 2.3. Rx-Coil Induced Voltage

Since the sensor can be modeled as a transformer, it is obvious to calculate also a transformation ratio between the Tx- and Rx-coils which yields

(5) UTxURx=MTx,TarITar−LTxITxMRx,TarITar+jRTxITxωMRx,TarITar︸≈0.

The imaginary part of this expression is set to zero, due to the negligibly small numerator compared to the denominator. Following, the induced voltage of the Rx-coils can be written as:

(6) $$U_{\mathrm{Rx}}=\frac{M_{\mathrm{Rx},\mathrm{Tar}}}{M_{\mathrm{Tx},\mathrm{Tar}}-L_{\mathrm{Tx}}\frac{I_{\mathrm{Tx}}}{I_{\mathrm{Tar}}}}\;\cdot \;U_{\mathrm{Tx}}\;.$$

As a result, the oscillator frequency is not directly included in Equation (6) and for the operating frequency range the self-inductance of the Tx-coil and the mutual inductances can be considered as constants. In good approximation, the induced voltage amplitude on the Rx-coils does not depend on the Tx-oscillator frequency.

### 2.4. Angular Error

Commonly, encoders are used with ambiguous sensor signals over 360° of mechanical rotation. This ambiguity is caused by the periodicity *p* of the encoder and leads to a distinction between the *mechanical* angle ϕ and the *electrical* angle $\phi_{p}$

(7) $$\phi_{p}=p\phi \;.$$

For the inductive coupled coils sensor the periodicity *p* equals the number of target wings. To calculate the encoded angle from *a* and *b*, the arctangent function is used. In case of ideal, undistorted signals, it yields:

(8) $$\mathrm{atan}\left(\frac{a(\phi )}{b(\phi )}\right)=\mathrm{atan}\left(\frac{\sin(p\phi )}{\cos(p\phi )}\right)=\mathrm{atan}\left(\tan(p\phi )\right)=\phi_{p}\;.$$

**Figure 4:**
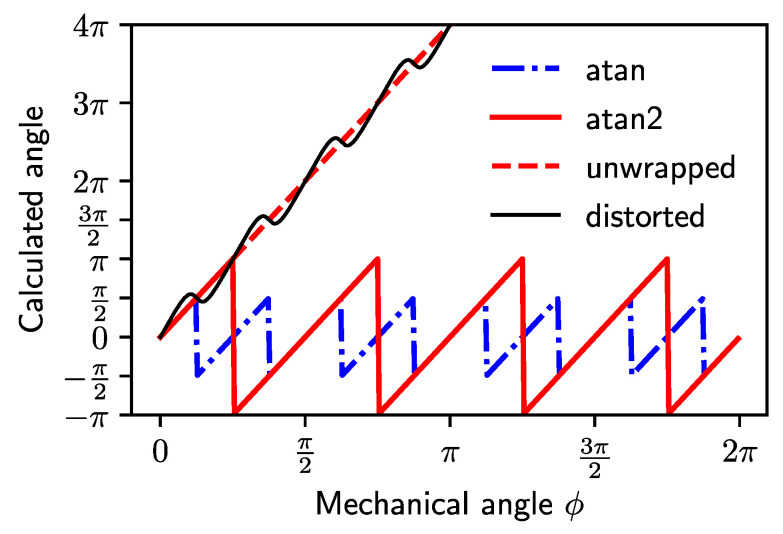
Calculated encoder angle in dependence of the mechanical reference angle ϕ for a sensor with periodicity p=4. For comparison, the angle is calculated by, atan2, and unwrapped atan2 functions. An exaggerated angular error deviating from the ideal output is shown as an example.

Equation (8) is only valid for a restricted range of pϕ because of quadrant ambiguities and eventual division by zero. In practice, this problem is solved by a modified two-argument arctangent function well known as atan2(a,b) (Figure 4).

Every encoder is influenced by undesired disturbances, which will cause an angular error. The electrical angular error be defined as the difference from the electrical reference angle

(9) $$\Delta \phi_{p}=\mathrm{atan}\left(\frac{a(\phi )}{b(\phi )}\right)-p\phi \;.$$

With respect to Equation (7), the mechanical angular error is defined as

(10) $$\Delta \phi =\frac{1}{p}\Delta \phi_{p}\;.$$

This shows the main benefit of a periodicity p>1: The angular error over a mechanical rotation decreases for higher periodicities *p*. However, the absolute angle can no longer be determined unambiguously without further measures.

## 3. Parameter Definition and Model Simplification

The first step for building up a general applicable simulation model is the parametrization of the sensor geometry. Since the Rx-coil is the most decisive element, the definition focuses on its dimensions. Due to the concentric arrangement of the coils, the dimensions of the Tx-coil and target following implicitly. Regarding the simplification, the Rx-coils are discarded from the simulation model. This way of simplifying the model is valid, since there is only neglectable coupling between the Tx- and Rx-coils (see Equation (3)). However, with the numerical implementation of Faraday’s law, results for the induced voltages can still be obtained.

### 3.1. Definition of the Sensor Geometry

**Figure 5:**
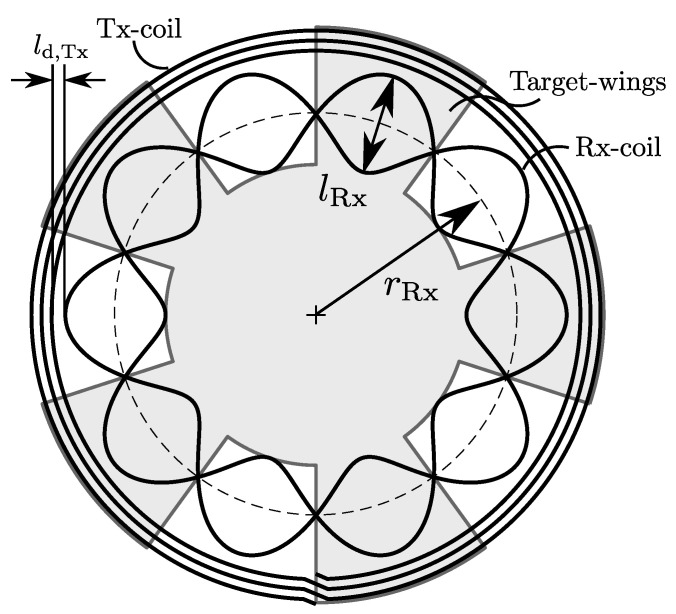
Sensor geometry shown as an example with periodicity p=5. In the radial dimension, the intermediate radius $r_{\mathrm{Rx}}$ and the peak-to-peak distance $l_{\mathrm{Rx}}$ of the Rx-coil define primarily the overall dimensions. The radii of the Tx-coil and target follow implicitly. Note that this is a simplified sketch, containing neither coil connections or vias.

Figure 5 shows the parametrization of the sensor geometry. The dimensions of the model are mostly dependent on the Rx-coil radius $r_{\mathrm{Rx}}$ and the peak-to-peak distance $l_{\mathrm{Rx}}$. For the Tx-coil and the target follows a implicit definition, since the inner radius of the Tx-coil is defined as

(11) $$r_{i,\mathrm{Tx}}=r_{\mathrm{Rx}}+l_{\mathrm{Rx}}/2+l_{d,\mathrm{Tx}}\;,$$

where $l_{d,\mathrm{Tx}}$ is a constant distance between the Rx- and Tx-coil. For the inner radius of the target follows

(12) $$r_{i,\mathrm{Tar}}=r_{\mathrm{Rx}}-l_{\mathrm{Rx}}/2\;.$$

The outer radius of the Tx-coil depends on the number of windings $n_{w}$ and the constant copper-width $l_{w,\mathrm{Cu}}$:

(13) $$r_{o,\mathrm{Tx}}=r_{i,\mathrm{Tx}}+\left(2n_{w}-1\right)\;\cdot \;l_{w,\mathrm{Cu}}\;,$$

whereby the outer Target radius equals the outer Tx-coil radius

(14) $$r_{o,\mathrm{Tar}}=r_{o,\mathrm{Tx}}\;.$$

In the following observations the distance between the Rx- and Tx-coil is set to $l_{d,\mathrm{Tx}}=0.774\;mm$ and the copper-width to $l_{w,\mathrm{Cu}}=0.254\;mm$.

### 3.2. Numeric Integration of Field Data

A numerical implementation of Faraday’s law is presented, to calculate the induced voltage of the polar-shaped Rx-coils from simulated B-field data. The simulation software of choice is *Ansys Maxwell3D* whereby the eddy current solver is used. This solver provides a steady-state solution which neglects wave propagation effects. All currents inside a conductor oscillate with the same user-defined frequency and there is no change in the electric phase angle along a conductor. The defined excitation is sinusoidal, which leads to a complex notation of all quantities. As a result of the simulation, the B-field data will be exported in complex notation on a defined grid in the 3D-space [10,11,12].

The first step is to find a mathematical description of the spanned Rx-surface *S*, because this is essential for the magnetic flux calculation. As shown in Figure 5, each Rx-coil consists of a positive and negative sine or cosine contour. It is necessary to place each Rx-coil on two PCB-layers in order to be able to use the same layers for both coils. This leads to jumps at every half period of the positive and negative contour (via-positions in Figure 1). Following this, the centerline of the Rx-coil tracks can be defined in cylindrical coordinates for every half period as

(15) $${\vec{C}}_{+/-}:=\left\{\left(\varphi ,z_{1},z_{2}\right)\in R\;|\;0\leq \varphi \leq \pi /p\;\mathrm{and}\;z_{2}>z_{1}\right\}\;,$$

which yields for the coordinates

(16) $${\vec{C}}_{+/-}=\left[\begin{array}{c} r_{\mathrm{Rx}}\pm {ł}_{\mathrm{Rx}}/2\;\cos\left(p\varphi \right)\\ \varphi \\ z_{1/2} \end{array}\right].$$

**Figure 6:**
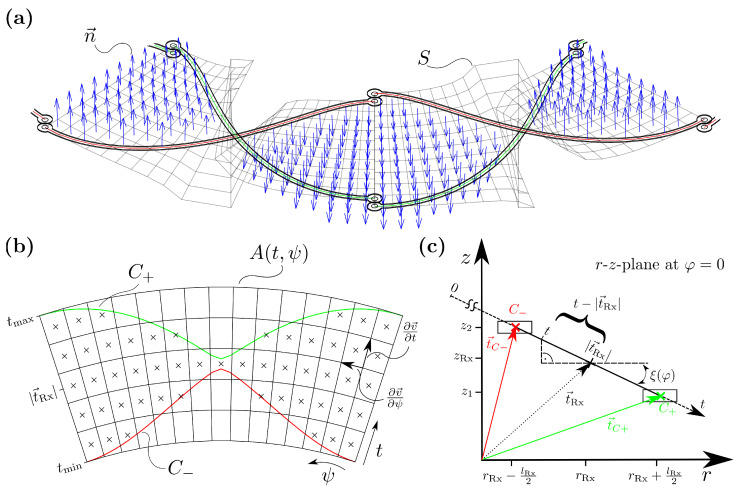
Surface definition for numeric integration of simulated B-field data for the Rx-coils. A surface-grid is defined, whereby only elements inside the boundaries—determined by the centerlines $C_{+}$ and $C_{-}$ of the Rx-coil tracks—contribute to the overall magnetic flux. The 3D-view of a cutout of the Rx-coil with the surface normals for the relevant elements is displayed in (**a**). Due to the complex geometry of the coil with crossing tracks on different layers, the surface rotates around the intermediate radius ${\vec{t}}_{\mathrm{Rx}}$. Accordingly, a parametrization of the unwound surface A(t,ψ) is introduced. (**b**) shows the discretization of this surface with low resolution for better illustration. The crossed surface-elements lie inside the integration boundaries and contribute therefore to the total magnetic flux. For the transformation in (t,ψ) coordinates, the inclination angle ξ for every position at angle φ needs to be known. (**c**) shows the cross-section in the *r*–*z*-plane. The calculation of ξ and the following parametrization requires only simple trigonometric expressions as shown in Equations (21) and (20).

As shown in Figure 6, the spanned surface rotates around the intermediate radius $r_{\mathrm{Rx}}$. A parametrization of the surface is introduced, depending on the curvilinear coordinates (t,ψ). These coordinates defining the position on the unwound surface *A* (Figure 6b)

(17) $$A:=\left\{\left(t,\psi \right)\in R^{2}\;|\;t_{\min}\leq t\leq t_{\max}\;\mathrm{and}\;0\leq \varphi \leq \pi /p\right\}\;,$$

where *t* denotes the parameter in the interpolated direction between $C_{+}$ and $C_{-}$ as well as the second parameter ψ, which equals the angular direction in cylindrical coordinates. The inner and outer limit of *A* is given by

(18) $$\begin{array}{c} \begin{array}{cc} t_{\min/\max} & =|{\vec{t}}_{\mathrm{Rx}}|\mp \frac{1}{2}\left|\;{\vec{t}}_{C+}\left(\varphi =0\right)-{\vec{t}}_{C-}\left(\varphi =0\right)\;\right|\\ \; & =\sqrt{r_{\mathrm{Rx}}^{2}+z_{\mathrm{Rx}}^{2}}\mp \frac{1}{2}\sqrt{l_{\mathrm{Rx}}^{2}+{\left(z_{1}-z_{2}\right)}^{2}}\;, \end{array} \end{array}$$

while for the intermediate z-coordinate follows

(19) $$z_{\mathrm{Rx}}=\frac{z_{1}+z_{2}}{2}\;$$

and the inclination angle around the intermediate radius, as shown in Figure 6c:

(20) $$\xi \left(\psi \right)=\mathrm{atan}\left(\frac{C_{+z}\left(\psi \right)-C_{-z}\left(\psi \right)}{C_{+r}\left(\psi \right)-C_{-r}\left(\psi \right)}\right)\;.$$

Now the complete parametrization is given with the vector $\vec{v}$, which maps every point at (t,ψ) to the corresponding point in cylindrical coordinates

(21) $$\vec{v}\left(t,\psi \right)=\left[\begin{array}{c} r(t,\psi )\\ \varphi (t,\psi )\\ z(t,\psi ) \end{array}\right]=\left[\begin{array}{c} \left(t-\right|{\vec{t}}_{\mathrm{Rx}}\left|\right)\cos\left(\xi \left(\psi \right)\right)+r_{\mathrm{Rx}}\\ \psi \\ \left(t-\right|{\vec{t}}_{\mathrm{Rx}}\left|\right)\sin\left(\xi \left(\psi \right)\right)+z_{\mathrm{Rx}} \end{array}\right]\;.$$

With a suitable parametrization of the spanned surface, the magnetic flux can be calculated

(22) $$\begin{array}{c} \begin{array}{cc} \Phi & =\underset{S}{\;\int \int \;}\vec{B}\;\cdot \;d\vec{S}=\underset{S}{\;\int \int \;}\left(\vec{B}\;\cdot \;\vec{n}\right)\;dS\\ \; & =\underset{A}{\;\int \int \;}\vec{B}\left(\vec{v}\left(t,\psi \right)\right)\;\cdot \;\underset{=\vec{n}}{\underset{︸}{\left(\frac{\partial \vec{v}}{\partial t}\times \frac{\partial \vec{v}}{\partial \psi }\right)}}\;t\;dt\;d\psi \end{array}\;, \end{array}$$

whereby the normal vectors of each surface element are calculated by a cross-product of the partial derivatives of $\vec{v}$ in *t*- and ψ-direction. For the numerical implementation, a weighting function *W* is needed, which sets the normal vectors of all surface elements outside the boundaries to zero [13]

(23) $$W\left(t^{\prime },\psi^{\prime }\right)=\left\{\begin{array}{cc} 1, & {\vec{C}}_{-}\leq {\vec{v}}^{\prime }\left(t^{\prime },\psi^{\prime }\right)\leq {\vec{C}}_{+}\\ 0, & \mathrm{otherwise} \end{array}\right.\;.$$

Within the definition of this function the notation $\left(t^{\prime },\psi^{\prime }\right)$ emphasizes that the relevant points for this case selection are not the grid points, but rather the centerpoints of the surface elements.

After the projection of the simulated B-field values $\vec{B}$ on the with *W* weightened normal vectors $\vec{n^{\prime }}$, only flux values for surface elements inside the integration boundaries are obtained, as illustrated in Figure 6a. Finally, the induced voltage can be calculated by a summation of the magnetic fluxes of each element and consideration of the angular frequency ω:

(24) $$U_{\mathrm{Rx}}=-j\omega \;\Phi =-j\omega \sum \vec{B}\;\cdot \;\vec{n^{\prime }}\;.$$

Instead of rotating the target in the simulation model, the integration boundaries will be rotated in the numerical calculation of Faraday’s law. Accordingly, only one simulation is needed for the calculation of one electrical period of the induced voltages. Since the numerical integration is much faster than the simulation of the full coil system, this leads to a huge benefit regarding computational time. Note, that this approach is valid as long as the field distribution caused by the Tx-coil and target is rather symmetrical in the angular direction.

## 4. Applied Statistical Methods

Instead of simulating each new coil design mathematical models are used, which will be able to predict various sensor quantities with less computational and time effort. Since this approach is based on linear regression models, simulation data are still once needed for the model calculation. However, this effort will be comparably small, because only a limited number of simulations is required.

In statistics the independent variables are commonly called *factors*, but in the following the term design parameters will further be used. Within the study, the parameters will be varied in defined steps. Due to the different parameter ranges, the steps will be normalized from −1 to +1. The amount of needed steps per parameter is k+1, where *k* is the highest order of the regression polynomial. A trivial approach is to calculate all parameter combinations. In the field of design of experiments this approach is known as *full-factorial* design. The goal here will be to minimize the number of experiments with a maximum informational output [14].

### 4.1. Linear Regression

The classical linear regression model is defined as [14]

(25) $$\vec{y}=X\;\cdot \;\vec{\beta }+\vec{\epsilon }\;,$$

where $\vec{y}$ is the dependent or *response variable*

(26) $$\vec{y}=\left[\begin{array}{c} y_{1}\\ \vdots \\ y_{m} \end{array}\right]\;,$$

X denotes the *design matrix* of order m×(n+1)

(27) $$X=\left[\begin{array}{cccc} 1 & x_{11} & \cdots & x_{1n}\\ \vdots & \vdots & \ddots & \vdots \\ 1 & x_{m1} & \cdots & x_{mn} \end{array}\right]\;,$$

$\vec{\beta }$ corresponds to the *regression coefficients*

(28) $$\vec{\beta }=\left[\begin{array}{c} \beta_{1}\\ \vdots \\ \beta_{n} \end{array}\right]$$

and $\vec{\epsilon }$ represents the *disturbance* terms

(29) $$\vec{\epsilon }=\left[\begin{array}{c} \epsilon_{1}\\ \vdots \\ \epsilon_{m} \end{array}\right]\;.$$

The response values $\vec{y}$ will be obtained by simulation in dependence of the respective parameter values in X. Subsequently, the regression coefficients can be calculated by the *least-squares method* with

(30) $$\vec{\epsilon }\;\cdot \;{\vec{\epsilon }}^{\;T}=\sum {\vec{\epsilon_{i}}}^{2}=\min\;,$$

which leads to

(31) $$\vec{\beta }={\left(X^{T}X\right)}^{-1}X^{T}\vec{y}\;.$$

Additionally, the linear regression model is based on the following assumptions [14]:

1. For the expectation value of the disturbance term follows $E(\vec{\epsilon })=0$
2. The data need to be homoscedastic with $\mathrm{Cov}\left(\vec{\epsilon }\right)=\sigma^{2}I$
3. The factors needs to be linearly independent shown by rk(X)=n

Note that for higher-order polynomials the regression will still be linear, since some matrix entries $x_{ij}$ will be substituted by a factor of order *k*.

### 4.2. D-Optimal Design

In the following, a short overview about the theory behind D-optimal designs will be given. The term *design* stands for a set of experiments—or in this case simulations—which should be reduced to a minimum. Accordingly, optimization means in this context the minimization of the design. Starting from a candidate set including all combinations of the parameter levels (full-factorial), the experiment should be limited to a number of *N* runs. As an criterion for optimization, D-optimal experiments minimize the covariance of the regression coefficients which is given by [15]

(32) $$\min\left(\Sigma_{\beta }\right)=\min\left(\mathrm{Cov}(\vec{\beta })\right)=\min\left(\sigma^{2}{\left(X^{T}X\right)}^{-1}\right)\;.$$

Since the determinant $\det(X^{T}X)$ is inversely proportional to the covariance in Equation (32), a *row-exchange* algorithm is used—similar to the method presented by Fedorov (pp. 104–110)—to maximize this determinant [16]

(33) $$\max\left(\det(X^{T}X)\right)\propto \min\left(\sigma^{2}{\left(X^{T}X\right)}^{-1}\right)\;.$$

In the end, the algorithm chooses those *N* experiments of the candidate set, which lead to *D-optimality*. It should be noted, that the term $X^{T}X$ is known as *Fisher information matrix* [14].

By means of considering the normal distribution of two random variables, the relationship between the covariance of the regression coefficients and the determinant of the Fisher information matrix can be shown. In this regard, one important realization is the similarity between the multivariate normal distribution and an ellipsoid [17].

Ellipsoids can be mathematically described by a *quadric*, which is the solution set of a quadratic equation. Generally spoken, a quadric ranks as an algebraic variety. For an ellipsoid it yields

(34) $${\left(\vec{x}-\vec{q}\right)}^{T}D\left(\vec{x}-\vec{q}\right)=1\;,$$

where $\vec{x}$ is the vector of the independent variables, $\vec{q}$ is the center-point vector and D a positive definite matrix. Additionally, the eigenvalues $\lambda_{Di}$ of the matrix D are related to the semi-axes $a_{i}$ of the ellipsoid described by Equation (34) as follows [17]

(35) $$\left[\begin{array}{c} \lambda_{D1}\\ \vdots \\ \lambda_{Di} \end{array}\right]=\left[\begin{array}{c} 1/a_{D1}^{2}\\ \vdots \\ 1/a_{Di}^{2} \end{array}\right]\;.$$

**Figure 7:**
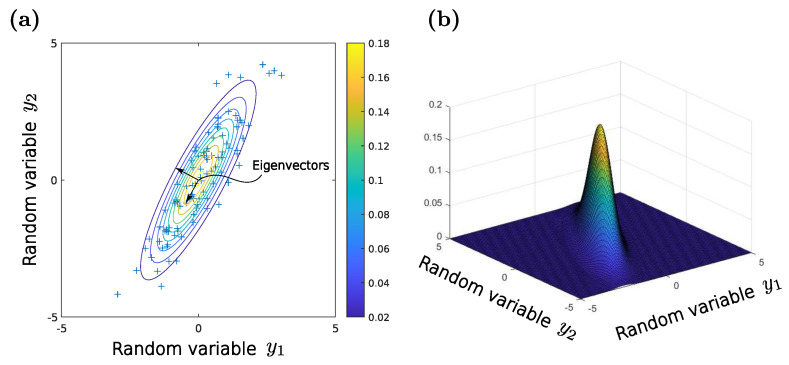
Example of two correlated random variables $y_{1}$ and $y_{2}$. The random variables are jointly normally distributed which is shown by the contours (**a**) of the underlying bivariate normal distribution (**b**). Accordingly, this plot elucidates the similarity to an ellipsoid, whereat the eigenvectors lying in the direction of the semi-axes.

Figure 7 shows a dataset following the distribution

(36) $$N\left(\vec{\mu },\Sigma \right)=N\left(\left[\begin{array}{c} 0\\ 0 \end{array}\right],\left[\begin{array}{cc} 1 & 1.5\\ 1.5 & 3 \end{array}\right]\right)\;.$$

An ellipsoid corresponding to this bivariate distribution can be described by [17]

(37) $${\left(\vec{x}-\vec{\mu }\right)}^{T}\Sigma^{-1}\left(\vec{x}-\vec{\mu }\right)=1\;.$$

Under the assumption of a positive definite matrix Σ the terms

(38) $$\det\left(\Sigma \right)=\frac{1}{\det(\Sigma^{-1})}$$

are identical, which leads—associated with Equation (35)—to

(39) $$\det\left(\Sigma \right)=\prod_{i=1}\lambda_{\Sigma i}=\prod_{i=1}a_{\Sigma i}^{2}\;.$$

This shows that the determinant of the covariance—or in respect to Equation (32) the determinant $\det(X^{T}X)$—is proportional to the size of the describing ellipsoid and therefore a good criterion for optimizing the design. Note that a D-optimal design needs at least n+1 experiments, where *n* is the number of regression coefficients.

## 5. D-Optimal Designs in Application

As mentioned in the introduction, regression models for the Tx-coil inductance, the induced voltage on the Rx-coils and the angular error are presented by generating D-optimal designs. For all models the highest order will be quadratic and only linear interaction terms are considered. With 6 parameters, this approach yields a model containing 28 terms:

(40) $$\begin{array}{c} \begin{array}{cc} \tilde{y}= & \beta_{0}+\beta_{1}r_{\mathrm{Rx}}+\beta_{2}l_{\mathrm{Rx}}+\beta_{3}n_{w}+\beta_{4}n_{l}+\beta_{5}h_{a}+\beta_{6}p+\beta_{12}r_{\mathrm{Rx}}l_{\mathrm{Rx}}+\beta_{13}r_{\mathrm{Rx}}n_{w}+\beta_{14}r_{\mathrm{Rx}}n_{l}+\beta_{15}r_{\mathrm{Rx}}h_{a}\\ \; & +\beta_{16}r_{\mathrm{Rx}}p+\beta_{23}l_{\mathrm{Rx}}n_{w}+\beta_{24}l_{\mathrm{Rx}}n_{l}+\beta_{25}l_{\mathrm{Rx}}h_{a}+\beta_{26}l_{\mathrm{Rx}}p+\beta_{34}n_{w}n_{l}+\beta_{35}n_{w}h_{a}+\beta_{36}n_{w}p+\beta_{45}n_{l}h_{a}\\ \; & +\beta_{46}n_{l}p+\beta_{56}h_{a}p+\beta_{11}r_{\mathrm{Rx}}^{2}+\beta_{22}l_{\mathrm{Rx}}^{2}+\beta_{33}n_{w}^{2}+\beta_{44}n_{l}^{2}+\beta_{55}h_{a}^{2}+\beta_{66}p^{2}\;. \end{array} \end{array}$$

Since the highest order of this polynomial is 2, at least three steps per parameter are needed. Following, a full-factorial design would require $3^{6}=729$ experiments. In contrast, a D-optimal design requires at least n+1=28 experiments.

A commonly used indicator for comparing different designs—especially with a different amount of experiments—is the *D-efficiency* which is calculated as [18]

(41) $$D_{\mathrm{eff}}=\frac{\det{\left(X^{T}X\right)}^{1/(n+1)}}{m}\;.$$

For an orthogonal design matrix the determinant $\det(X^{T}X)$ gets equal to $m^{\left(n+1\right)}$. Hence, the D-efficiency would be 100% for such a design.

### 5.1. Tx-Inductance Model

**Figure 8:**
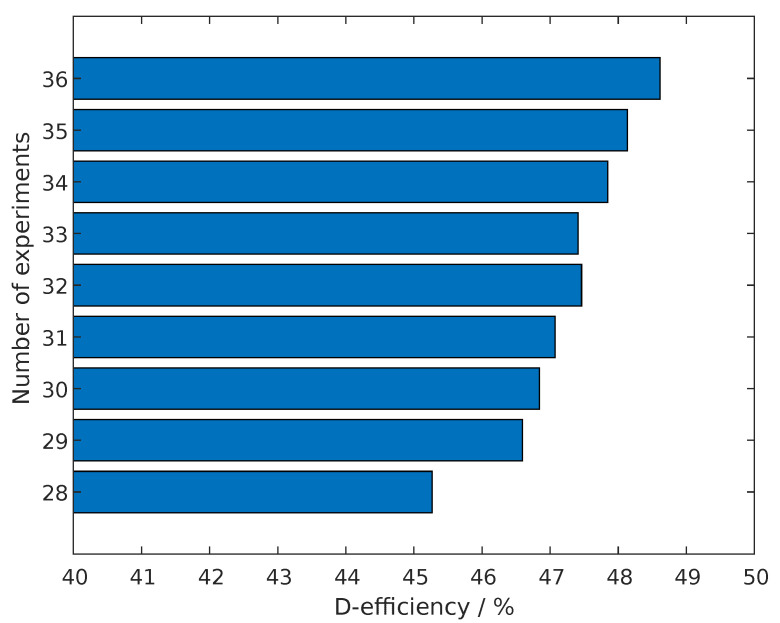
Comparision of the D-efficiency of designs with different number of experiments. The compared designs containing the terms of Equation (40).

The first regression model gives an estimation for the Tx-coil inductance in the presence of a target. The polynomial will be dependent on the variables listed in Table 1. In order to find a good compromise between simulation time and informational output, the D-efficiency (according to Equation (41)) is compared for different numbers of experiments in Figure 8. A number of N=32 runs will be chosen, since the D-efficiency is comparatively high with over 47% and the available computational resources allow to run 16 simulations in parallel.

Table A1 contains the result of the row-exchange algorithm in normalized notation. Each experiment represents one simulation. Hence, the result will be 32 inductance values which will be fitted. According to monotonous increasing residuals in a first fit, the simulated inductance values are logarithmized. This is a common transformation and leads finally to an exponential model [19].

**Figure 9:**
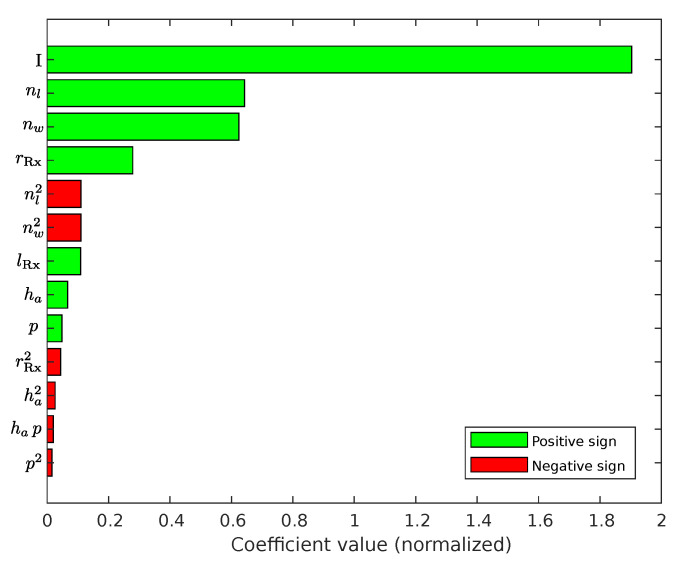
Pareto-diagram related to the normalized regression coefficients of the Tx-coil inductance model. It shows the 13 most significant coefficients in descending order. The term denoted by *I* represents the identity term, which is related to $\beta_{0}$. Please note that those values correspond to the exponential model as shown in Equation (42).

Figure 9 shows the values of the 13 most significant regression coefficients in descending order. Those values showing the effects of the actual terms, since the regression coefficients can also be interpreted as a weighting factor. In this case, the number of windings $n_{w}$, number of layers $n_{l}$ and intermediate radius $r_{\mathrm{Rx}}$ have the biggest effect on the Tx-coil inductance. Since the absolute values of the coefficients dropping after the 13th term rapidly, the model will be reduced to

(42) $$\begin{array}{c} \begin{array}{cc} {\tilde{L}}_{\mathrm{Tx}}= & \exp(-5.72+107.83\;m^{-1}\;r_{\mathrm{Rx}}+43.62\;m^{-1}\;l_{\mathrm{Rx}}+1.28\;n_{w}+1.31\;n_{l}+170.54\;m^{-1}\;h_{a}+29.5010^{-3}\;p\\ \; & -4.95\;m^{-1}\;h_{a}p-1.7410^{3}\;m^{-2}\;r_{\mathrm{Rx}}^{2}-109.7010^{-3}\;n_{w}^{2}-110.8010^{-3}\;n_{l}^{2}-26.4610^{3}\;m^{-2}\;h_{a}^{2}\\ \; & -1.010^{-3}\;\;p^{2})\mu H. \end{array} \end{array}$$

The regression coefficients of the full model are listed in Table A2. In Equation (42) the regression coefficients have been transformed in order to allow unnormalized parameter levels (Table 1) inside the equation.

**Figure 10:**
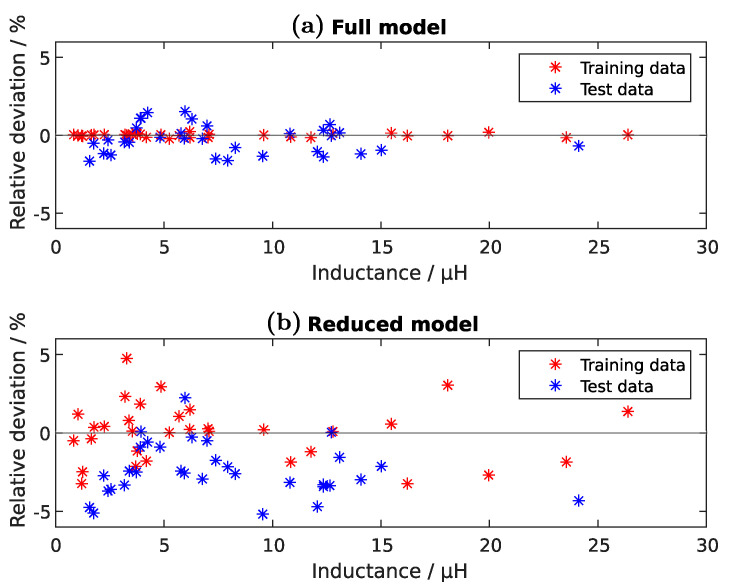
Relative deviation between the training or test data and the regression model of the Tx-coil inductance. The full-model (**a**)—considering all coefficients from Equation (40)—is compared with the reduced model (**b**) from Equation (42). The training data respond to the experiments of Table A1. The test dataset contains also 32 experiments with randomly generated parameter levels inside the boundaries listed in Table 1.

Additionally to the dataset of Table A1, a dataset with 32 random parameter levels is generated, but the values lie still inside the boundaries of Table 1. This dataset will be denoted as *test data* and is not considered for the calculation of the regression model (see Table A3). Figure 10 shows the relative deviation between the simulated value and the estimation given by the regression model from Equation (42). The predicted inductance values of Equation (42) are in excellent agreement with the simulated results, since the deviation range is only between approximately −2%to+2% for the full model and between −5%to+5% for the reduced model. The very low deviation in case of the training data shows that the chosen exponential polynomial leads to a good representation of the physical behavior.

### 5.2. Rx-Voltage Amplitude Model

According to the chosen parametrization of the sensor geometry, the same simulation models and design matrix can be used for the regression model of the Rx-coil voltage amplitude. The proposed numerical integration method is applied to the exported field data of the simulated models. It is necessary to calculate only the induced voltage amplitude for one coil due to symmetry. However, the mesh resolution of the FE simulation has to be high enough in the vacuum region of the Rx-coils. A coarse mesh could lead to numerical inaccuracy, especially in further analysis whereby the integration boundaries are shifted for the representation of a target rotation.

**Figure 11:**
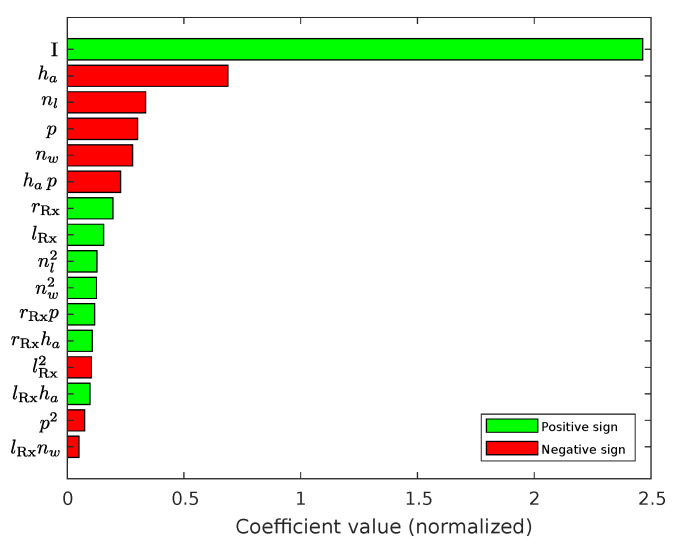
Pareto-diagram related to the normalized regression coefficients of the Rx-voltage amplitude model. It shows the 16 most significant coefficients in descending order. The term denoted by *I* represents the identity term, which is related to $\beta_{0}$. Please note that those values correspond to the exponential model as shown in Equation (43).

Compared to the Tx-coil inductance model, the coefficient values in Figure 11 are not decreasing as rapidly after the four most significant terms. Besides the identity term, the air gap $h_{a}$ has the highest effect to the Rx-voltage amplitude. This is expected because the magnetic field strength decreases exponentially with the distance from the source. Interestingly, the next significant terms are not the parameters $r_{\mathrm{Rx}}$ and $l_{\mathrm{Rx}}$ who determine the radial dimension, but rather the number of windings $n_{w}$ and layers $n_{l}$ together with the periodicity *p* of the sensor. As shown with Figure 9, $n_{w}$ and $n_{l}$ have a high influence on the Tx-coil inductance. Correspondingly, the Tx-coil inductance has also an high impact on the Rx-voltage amplitude, since the Tx-current will decrease with an increasing inductance value. An explanation for the effect of the parameter *p* is the impact on the spanned Rx-coil surface, which can decrease for high periodicities. This is related to the polar sine geometry of the Rx-coils and the increasing slope for higher periodicities, which leads to less coverage of the available area.

Analogously to the Tx-coil inductance model, the model for the Rx-voltage amplitude is reduced to the significant terms displayed in Figure 11:

(43) $$\begin{array}{c} \begin{array}{cc} {\tilde{U}}_{\mathrm{Rx}}= & \exp(6.37-22.25\;m^{-1}\;r_{\mathrm{Rx}}+313.41\;m^{-1}\;l_{\mathrm{Rx}}-882.00\times 10^{-3}\;n_{w}-1.10\;n_{l}-1.02\times 10^{3}\;m^{-1}\;h_{a}\\ \; & -30.70\times 10^{-3}\;p+21.40\times 10^{3}\;m^{-2}\;r_{\mathrm{Rx}}h_{a}+5.86\;m^{-1}\;r_{\mathrm{Rx}}p-20.11\;m^{-1}\;l_{\mathrm{Rx}}n_{w}+38.00\times 10^{3}\;m^{-2}\;l_{\mathrm{Rx}}h_{a}\\ \; & -57.07m^{-1}\;h_{a}p-16.60\times 10^{3}m^{-2}\;l_{\mathrm{Rx}}^{2}+125.40\times 10^{-3}\;n_{w}^{2}+127.20\times 10^{-3}\;n_{l}^{2}-4.70\times 10^{-3}\;p^{2})\;mV. \end{array} \end{array}$$

In Equation (43) the regression coefficients have been transformed in order to allow unnormalized parameter levels (Table 1) inside the equation.

**Figure 12:**
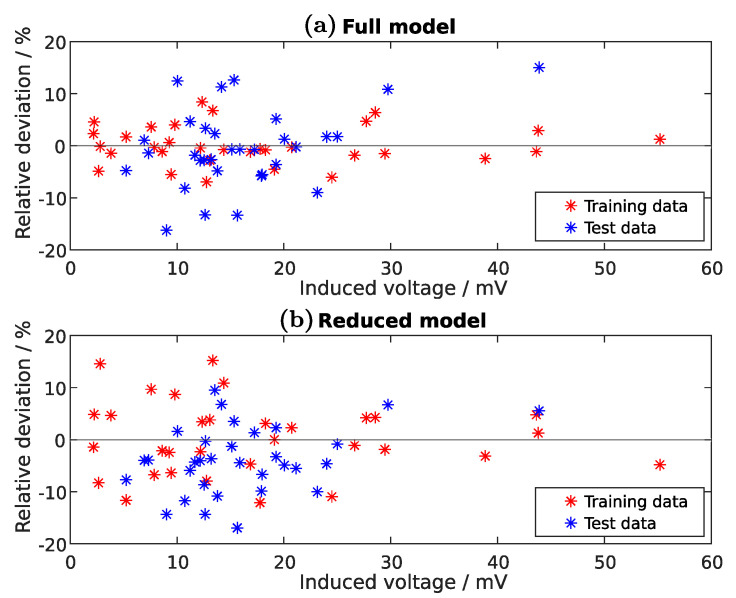
Relative deviation between the training or test data and the regression model of the Rx-voltage amplitude. The full-model (**a**)—considering all coefficients from Equation (40)—is compared with the reduced model (**b**) from Equation (43). The training data respond to the experiments of Table A1. The test dataset contains also 32 experiments with randomly generated parameter levels inside the boundaries listed in Table 1.

The prediction is less accurate as for the Tx-coil inductance model, but gives still an acceptable result (Figure 12). The relative deviations for the full and reduced models lie between −17%to+15%. Note, that this model relates to a driving voltage amplitude of $U_{\mathrm{Tx}}=1\;V$, which is linearly related to the Rx-voltage amplitude as Equation (6) shows. For different driving voltages the result of Equation (43) can be scaled.

### 5.3. Angular Error Model

The angular error is a special case, since not only the maximum value is of high interest, but the harmonic composition. In order to predict this behavior a description in form of a Fourier series is introduced with

(44) $$\tilde{\Delta \phi_{p}}=\tilde{H_{0}}+\sum_{k=1}^{5}\tilde{H_{k}}\cos\left(k\phi_{p}+\tilde{\delta_{k}}\right)\;,$$

where $\tilde{H_{k}}$ denotes the amplitude and $\tilde{\delta_{k}}$ the phase of the harmonic of order *k*. A regression polynomial will be calculated for each of those characteristic values, which leads to 11 models in total for a fit up to order k=5. Similarly to the other models, data will still be obtained by the numeric integration method, whereby now the signals over one electrical period are needed. Previously, this method was only applied for one target position or reference angle. As mentioned before, only one position is simulated per experiment and the *B-field* data are exported. The induced voltage signals over one electrical period can be obtained by iterative numeric integration with shifted integration boundaries in the angular direction. With the complete Rx-voltage signals, the electrical angle (Equation (8)) and electrical angular error (Equation (9)) can be calculated. Afterwards, a discrete Fourier transform (DFT) is applied to analyse the harmonic composition of the angular error and obtain the data for the regression analysis.

It turns out that the model approach of Equation (40) leads to an insufficient prediction accuracy. Therefore, the air gap $h_{a}$ is eliminated from the study, whereby the remaining parameters and levels still equals Table 1. The air gap has a high influence on the angular error, since the magnetic field distribution over the Rx-coils changes significantly with this parameter and in turn, the field distribution determines finally the angular error composition. Furthermore, the design needs to be recalculated due to the different number of parameters. The new design contains 25 experiments and needs to be evaluated for each air gap separately (Table A4).

The model approach equals Equation (40). Only the terms containing $h_{a}$ will be set to zero. The target values of the harmonic amplitudes $H_{k}$ will again be logarithmized due to exponential behavior, but the phase information $\delta_{k}$ and offset $H_{0}$ will still be linear. For readability reasons the whole model equations are not written down. The coefficient values can be obtained from the appendix.

**Figure 13:**
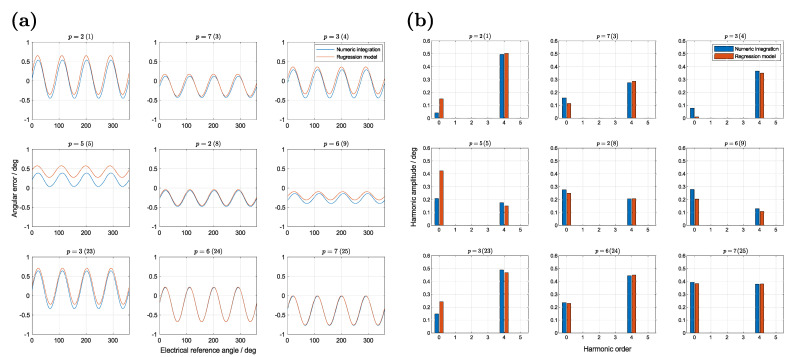
Comparison of the electrical angular error signal and harmonics of nine randomly chosen examples of the test dataset. For each harmonic of the orders 0 to 5 a regression model is calculated for the harmonic amplitude and phase. The right plots show the harmonic amplitudes (**b**), whereby the left plots show the total error curve (**a**) according to Equation (44). The data were calculated for an air gap of $h_{a}=0.5\;mm$ and the index of each plot title (in brackets) refers to Table A6.

Figure 13 shows nine randomly chosen examples of the test dataset for an air gap $h_{a}=0.5\;mm$. The left side contains the composed angular error from the regression models, as written in Equation (44). Accordingly, the right side shows only the agreement between the values obtained by numeric integration and the regression models for the harmonic amplitudes. Observing the results, only 0th and 4th order harmonics are present in the angular error. Harmonics of other orders are numerically equal to zero. This is related to the simplified simulation model, which contains only the idealized coil system without any further copper-tracks of a PCB. Considering the prediction accuracy, the mostly dominant 4th harmonic is well fitted with a relative deviation in the range of −23%to+11%. In some cases, the deviation of the offset is huge with up to ±260% for very small absolute values. For most applications this is acceptable, since an offset of the angular signal can be compensated.

Table 2 shows exemplarily the comparison for model no. 20 of Table A4. A step size of 1deg is chosen, which leads in summary to 72 individual simulations. In the parallelized case, 16 target positions were calculated simultaneously. If the available computational resources are limited, only a sequential calculation might be possible, where only one simulation runs at a time. With the simplified model and following numeric integration, only one target position needs to be simulated. To reduce discretization errors, the mesh resolution in the area where the Rx-coils would lie (vacuum) was increased. A lower mesh density could also lead to an acceptable solution accuracy and would require less memory. In the numeric integration, the Rx-surface was discretised in 10 steps in the radial and 180 steps in the angular direction. Regarding computational time, the simplified model is the fastest way of calculation. The additional effort for the following numeric integration is thereby negligible, since the calculation is done in under 10 s.

### 5.4. Validation with Measurements

**Figure 14:**
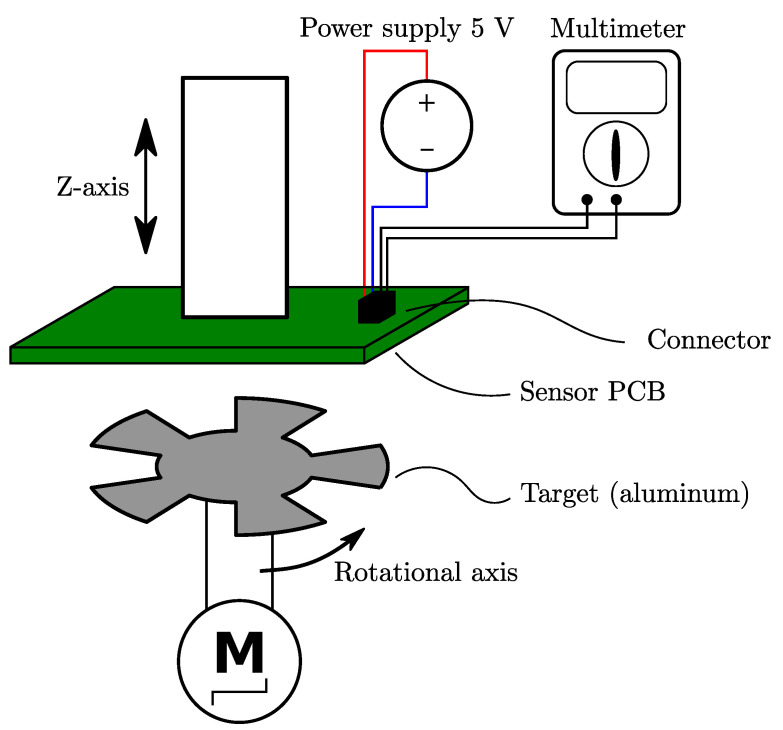
Overview about the test setup. The sensor PCB is mounted on a linear axis for adjusting the air gap. On the opposite side is the target. The rotation is performed by a stepper motor. This motor also includes an encoder system, which gives the reference angle for the measurement. The measurement procedure is quasi-static. The rotation will stop at each angle, while the output voltages are measured with a digital multimeter.

**Figure 15:**
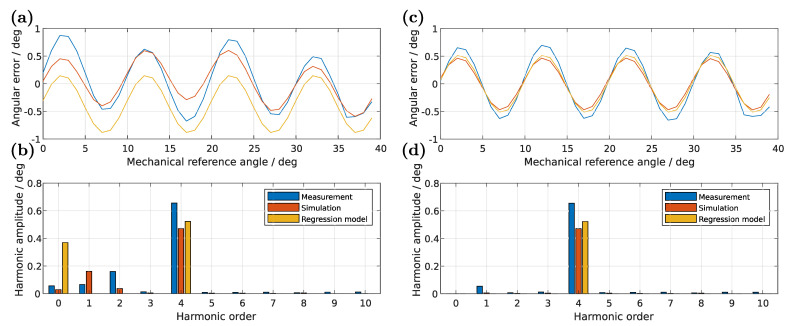
Comparison of the electrical angular error signals (**a**,**c**) and harmonics (**b**,**d**) between measurement, simulation containing the full PCB-layout and prediction of the presented regression models for experiment no. 4 of the training dataset for an air gap of 0.5mm. The left side (**a**,**b**) shows the raw results. On the right side (**c**,**d**) an amplitude-, offset- and orthogonality-correction was applied to the induced voltage signals and a mean compensation to the predicted angular error in case of the regression model.

As a last step, a proof of the presented models will be given by a measurement of a real sensor-system (see Figure 14). The measurement results will be compared with a simulation model containing the whole PCB-layout and the presented prediction models. Similar to the simulation, one angle is measured at a time without continuous movement of the target (quasi-static). The results of Table 3 are in good agreement with the previous test datasets. For the angular error in Figure 15 follows a good prediction of the dominant 4th harmonic, but a high deviation in the offset. Furthermore, also harmonics up to the 3rd order are present in the measurement and simulation, but cannot be obtained from the regression models. An reasonable explanation for the wrong prediction of these low order harmonics are the not-considered copper tracks of the PCB, since the simulation regarding the full layout is in good agreement with the measurement. However, as the right plots of Figure 15 shows, those harmonics can be eliminated by standard compensation methods—as proposed by Kuntz et al.—and therefore are not highly relevant [20].

## 6. Conclusions

A novel method for the modeling and simulation of inductive coupled-coil-based angle encoders was introduced to reduce computational effort and calculate regression models for the prediction of sensor quantities without even a FE simulation. The method contains four major steps, starting with the simplification and parametrization of the sensor geometry in order to obtain a generally applicable simulation model. By virtue of the physical sensor principle, the observations focus on six geometric parameters: the intermediate Rx-coil radius $r_{\mathrm{Rx}}$, the Rx peak-to-peak distance $l_{\mathrm{Rx}}$, the number of Tx-windings per layer $n_{w}$, the number of Tx-layers $n_{l}$, the air gap between coils and target $h_{a}$ and the periodicity of the sensor *p*. For the calculation of a regression model, each parameter needs to be varied in defined steps. For instance, 729 simulations would be required if three steps are considered per parameter. In order to reduce this effort, the next step in the proposed method is to find a *D-optimal design*, which reduces the number of simulations to N=32 or N=25 if the air gap is eliminated from the model.

At this point, data can be generated by simulation for the selected parameter variations. Due to the chosen parametrization, the same simulations can be used for the calculation of the Tx-coil inductance and the Rx-voltage amplitudes. Although the Rx-coils were excluded from the simulation model, the resulting induced voltages can still be obtained by a numerical implementation of Faraday’s law. Therefore, an integration method is presented, which is applied to the exported B-field data from the simulation. This method has its biggest benefit when it comes to the calculation of a full electrical period. Normally, this leads to multiple simulations, since every target position needs to be calculated separately. With this method only one simulation is required, since the induced voltages for every angle can be calculated from one field data file by shifting the integration boundaries in the angular direction.

Once the data are acquired, linear regression models can be fitted by the common least-squares method. The deviation between the predicted values from the calculated models and the simulated results were in the range of −2%to+2% for the Tx-inductance and from −17%to+15% for the Rx-voltage amplitudes. A model for the electrical angular error was also calculated, although this is a special case, since a definition of a Fourier series is needed in order to predict the complete signal behavior. As the results show, only 0th and 4th order harmonics appear in the electrical spectrum. The in most cases dominant 4th order harmonic can be predicted with a deviation in the range of −23%to+11%. Unfortunately, the prediction of the offset is less accurate with up to ±260%. However according to the measurement, the 0th harmonic can be compensated by simple correction methods. Correspondingly, the 4th is of higher relevance, since simple corrections do not have an impact.

As an outlook, further parameters could be included in the regression models or different parameter ranges might be investigated. Additionally, the applied methods in combination with the calculated regression polynomials leave space for optimization. It is conceivable that the application of optimization algorithms on those models also lead to new insights.

## Figures and Tables

**Table 1:** Parameters describing the sensor geometry. For each parameter three levels are required, since the maximum order of the regression polynomial is two.

Variable	Levels	Quantity
$r_{\mathrm{Rx}}$	[10,15,20]mm	Intermediate Rx-coil radius
$l_{\mathrm{Rx}}$	[5,7.5,10]mm	Rx-coil peak-to-peak distance
$n_{w}$	[2,3,4]	No. of Tx-windings per layer
$n_{l}$	[2,3,4]	No. of Tx-layers
$h_{a}$	[0.5,1.5,2.5]mm	Air gap
*p*	[1,5,9]	Periodicity

**Table 2:** Computational resources for no. 20 of Table A4 evaluated by a sequential and parallelized simulation of the full model over one electrical period and one simulation of the simplified model with following numeric integration.

	Sequential	Parallelized	Simplified
Comp. time	138h	9h35min	4h01min
Max. RAM	26.8GB	428.8GB	77.7GB
CPU cores	1	16	1

**Table 3:** Comparison between measurement results and prediction of the models according to Equations (42) and (43). A scaling factor of 1.2 was introduced to the model of the Rx-voltage amplitude, since the Tx driving-voltage amplitude equals 1.2V for the tested sample.

	Measurement	Model	Relative Deviation
$L_{\mathrm{Tx}}$	2.12μH	2.05μH	−3.30%
$U_{\mathrm{Rx}}$	67.61mV	71.13mV	+5.21%

## Data Availability

Not applicable.

## Appendix A. D-Optimal Design for the Tx-Inductance and the Rx-Voltage Amplitude

Table A1 shows the full design matrix in normalized notation and the response values used for the calculation of the regression models of the Tx-coil inductance and the Rx-voltage amplitude. The regression coefficients for the full models are listed in Table A2. The randomly generated test parameter levels with the corresponding response values can be obtained from Table A3.

**Table A1:** D-optimal design with 32 experiments. The parameter levels are normalized from −1 to +1. In the experiments those levels correspond to the values in Table 1.

No.	Design Matrix																												Training Data	
	I	$r_{\mathrm{Rx}}$	$l_{\mathrm{Rx}}$	$n_{w}$	$n_{l}$	$h_{a}$	p	$r_{\mathrm{Rx}}l_{\mathrm{Rx}}$	$r_{\mathrm{Rx}}n_{w}$	$r_{\mathrm{Rx}}n_{l}$	$r_{\mathrm{Rx}}h_{a}$	$r_{\mathrm{Rx}}p$	$l_{\mathrm{Rx}}n_{w}$	$l_{\mathrm{Rx}}n_{l}$	$l_{\mathrm{Rx}}h_{a}$	$l_{\mathrm{Rx}}p$	$n_{w}n_{l}$	$n_{w}h_{a}$	$n_{w}p$	$n_{l}h_{a}$	$n_{l}p$	$h_{a}p$	$r_{\mathrm{Rx}}^{2}$	$l_{\mathrm{Rx}}^{2}$	$n_{w}^{2}$	$n_{l}^{2}$	$h_{a}^{2}$	$p^{2}$	$\log(L_{\mathrm{Tx}}/\mu H)$	$\log(U_{\mathrm{Rx}}/\mathrm{mV})$
1	+1	+1	+1	−1	−1	−1	−1	+1	−1	−1	−1	−1	−1	−1	−1	−1	+1	+1	+1	+1	+1	+1	+1	+1	+1	+1	+1	+1	0.571	4.011
2	+1	−1	+1	0	0	+1	+1	−1	0	0	−1	−1	0	0	+1	+1	0	0	0	0	0	+1	+1	+1	0	0	+1	+1	1.739	0.805
3	+1	+1	+1	−1	−1	+1	+1	+1	−1	−1	+1	+1	−1	−1	+1	+1	+1	−1	−1	−1	−1	+1	+1	+1	+1	+1	+1	+1	0.807	2.571
4	+1	−1	−1	+1	−1	−1	+1	+1	−1	+1	+1	−1	−1	+1	+1	−1	−1	−1	+1	+1	−1	−1	+1	+1	+1	+1	+1	+1	1.162	2.906
5	+1	−1	−1	−1	+1	−1	+1	+1	+1	−1	+1	−1	+1	−1	+1	−1	−1	+1	−1	−1	+1	−1	+1	+1	+1	+1	+1	+1	1.187	2.878
6	+1	−1	+1	+1	−1	−1	−1	−1	−1	+1	+1	+1	+1	−1	−1	−1	−1	−1	−1	+1	+1	+1	+1	+1	+1	+1	+1	+1	1.263	3.383
7	+1	+1	−1	−1	−1	−1	+1	−1	−1	−1	−1	+1	+1	+1	+1	−1	+1	+1	−1	+1	−1	−1	+1	+1	+1	+1	+1	+1	0.499	3.776
8	+1	+1	−1	−1	+1	−1	−1	−1	−1	+1	−1	−1	+1	−1	+1	+1	−1	+1	+1	−1	−1	+1	+1	+1	+1	+1	+1	+1	1.657	3.198
9	+1	−1	−1	−1	−1	−1	−1	+1	+1	+1	+1	+1	+1	+1	+1	+1	+1	+1	+1	+1	+1	+1	+1	+1	+1	+1	+1	+1	−0.188	3.780
10	+1	0	0	+1	0	−1	+1	0	0	0	0	0	0	0	0	0	0	−1	+1	0	0	−1	0	0	+1	0	+1	+1	2.383	2.825
11	+1	+1	−1	+1	+1	+1	+1	−1	+1	+1	+1	+1	−1	−1	−1	−1	+1	+1	+1	+1	+1	+1	+1	+1	+1	+1	+1	+1	3.159	0.979
12	+1	0	+1	−1	+1	+1	0	0	0	0	0	0	−1	+1	+1	0	−1	−1	0	+1	0	0	0	+1	+1	+1	+1	0	1.822	2.153
13	+1	−1	+1	−1	−1	−1	+1	−1	+1	+1	+1	−1	−1	−1	−1	+1	+1	+1	−1	+1	−1	−1	+1	+1	+1	+1	+1	+1	0.184	3.660
14	+1	−1	−1	−1	−1	+1	+1	+1	+1	+1	−1	−1	+1	+1	−1	−1	+1	−1	−1	−1	−1	+1	+1	+1	+1	+1	+1	+1	0.032	1.030
15	+1	+1	+1	0	+1	−1	+1	+1	0	+1	−1	+1	0	+1	−1	+1	0	0	0	−1	+1	−1	+1	+1	0	+1	+1	+1	2.739	2.951
16	+1	+1	+1	+1	+1	+1	−1	+1	+1	+1	+1	−1	+1	+1	+1	−1	+1	+1	−1	+1	−1	−1	+1	+1	+1	+1	+1	+1	3.272	2.026
17	+1	−1	−1	+1	+1	+1	0	+1	−1	−1	−1	0	−1	−1	−1	0	+1	+1	0	+1	0	0	+1	+1	+1	+1	+1	0	2.548	0.780
18	+1	+1	0	0	−1	+1	0	0	0	−1	+1	0	0	0	0	0	0	0	0	−1	0	0	+1	0	0	+1	+1	0	1.432	2.501
19	+1	+1	−1	+1	−1	−1	−1	−1	+1	−1	−1	−1	−1	+1	+1	+1	−1	−1	−1	+1	+1	+1	+1	+1	+1	+1	+1	+1	1.580	3.281
20	+1	+1	+1	+1	−1	−1	+1	+1	+1	−1	−1	+1	+1	−1	−1	+1	−1	−1	+1	+1	−1	−1	+1	+1	+1	+1	+1	+1	1.950	3.352
21	+1	−1	−1	+1	−1	+1	−1	+1	−1	+1	−1	+1	−1	+1	−1	+1	−1	+1	−1	−1	+1	−1	+1	+1	+1	+1	+1	+1	1.216	2.063
22	+1	−1	0	+1	+1	−1	−1	0	−1	−1	+1	+1	0	0	0	0	+1	−1	−1	−1	−1	+1	+1	0	+1	+1	+1	+1	2.465	2.666
23	+1	−1	+1	+1	+1	0	+1	−1	−1	−1	0	−1	+1	+1	0	+1	+1	0	+1	0	+1	0	+1	+1	+1	+1	0	+1	2.786	1.340
24	+1	−1	+1	−1	+1	−1	−1	−1	+1	−1	+1	+1	−1	+1	−1	−1	−1	+1	+1	−1	−1	+1	+1	+1	+1	+1	+1	+1	1.324	3.322
25	+1	+1	−1	−1	0	+1	−1	−1	−1	0	+1	−1	+1	0	−1	+1	0	−1	+1	0	0	−1	+1	+1	+1	0	+1	+1	1.307	2.283
26	+1	−1	0	−1	+1	+1	−1	0	+1	−1	−1	+1	0	0	0	0	−1	−1	+1	+1	−1	−1	+1	0	+1	+1	+1	+1	1.360	2.247
27	+1	−1	+1	−1	−1	+1	−1	−1	+1	+1	−1	+1	−1	−1	+1	−1	+1	−1	+1	−1	+1	−1	+1	+1	+1	+1	+1	+1	0.215	3.031
28	+1	+1	−1	+1	+1	−1	0	−1	+1	+1	−1	0	−1	−1	+1	0	+1	−1	0	−1	0	0	+1	+1	+1	+1	+1	0	2.994	2.591
29	+1	0	−1	0	+1	0	−1	0	0	0	0	0	0	−1	0	+1	0	0	0	0	−1	0	0	+1	0	+1	0	+1	2.261	2.227
30	+1	0	+1	+1	−1	+1	+1	0	0	0	0	0	+1	−1	+1	+1	−1	+1	+1	−1	−1	+1	0	+1	+1	+1	+1	+1	1.822	1.654
31	+1	+1	0	−1	+1	0	+1	0	−1	+1	0	+1	0	0	0	0	−1	0	−1	0	+1	0	+1	0	+1	+1	0	+1	1.957	2.513
32	+1	+1	+1	+1	0	0	0	+1	+1	0	0	0	+1	0	0	0	0	0	0	0	0	0	+1	+1	+1	0	0	0	2.748	2.652

**Table A2:** Regression coefficients for the full model of the Tx-inductance and Rx-voltage amplitude. The coefficients are given in normalized notation. Hence, the parameter levels must be normalized from −1 to +1, according to the boundaries listed in Table 1. Note that those values were calculated for logarithmized data (exponential model).

Term	Regression Coefficient $\beta_{i}$	
	${\tilde{L}}_{\mathrm{Tx}}$	${\tilde{U}}_{\mathrm{Rx}}$
*I*	1.905	2.465
$r_{\mathrm{Rx}}$	$278.9\times 10^{-3}$	$195.8\times 10^{-3}$
$l_{\mathrm{Rx}}$	$109.1\times 10^{-3}$	$156.2\times 10^{-3}$
$n_{w}$	$624.5\times 10^{-3}$	$-280.5\times 10^{-3}$
$n_{l}$	$642.4\times 10^{-3}$	$-336.1\times 10^{-3}$
$h_{a}$	$66.5\times 10^{-3}$	$-689.1\times 10^{-3}$
*p*	$48.6\times 10^{-3}$	$-301.1\times 10^{-3}$
$r_{\mathrm{Rx}}l_{\mathrm{Rx}}$	$-10.2\times 10^{-3}$	$7.6\times 10^{-3}$
$r_{\mathrm{Rx}}n_{w}$	$-76.2\times 10^{-6}$	$5.1\times 10^{-3}$
$r_{\mathrm{Rx}}n_{l}$	$3.9\times 10^{-3}$	$-5.7\times 10^{-3}$
$r_{\mathrm{Rx}}h_{a}$	$3.0\times 10^{-3}$	$107.0\times 10^{-3}$
$r_{\mathrm{Rx}}p$	$3.3\times 10^{-3}$	$117.2\times 10^{-3}$
$l_{\mathrm{Rx}}n_{w}$	$3.4\times 10^{-6}$	$-50.3\times 10^{-3}$
$l_{\mathrm{Rx}}n_{l}$	$643.6\times 10^{-6}$	$-29.2\times 10^{-3}$
$l_{\mathrm{Rx}}h_{a}$	$-5.7\times 10^{-3}$	$97.2\times 10^{-3}$
$l_{\mathrm{Rx}}p$	$3.6\times 10^{-3}$	$3.2\times 10^{-3}$
$n_{w}n_{l}$	$10.9\times 10^{-3}$	$-37.1\times 10^{-3}$
$n_{w}h_{a}$	$2.5\times 10^{-3}$	$42.8\times 10^{-3}$
$n_{w}p$	$6.2\times 10^{-3}$	$6.7\times 10^{-3}$
$n_{l}h_{a}$	$-5.2\times 10^{-3}$	$4.5\times 10^{-3}$
$n_{l}p$	$-2.4\times 10^{-3}$	$21.1\times 10^{-3}$
$h_{a}p$	$-19.8\times 10^{-3}$	$-228.3\times 10^{-3}$
$r_{\mathrm{Rx}}^{2}$	$43.4\times 10^{-3}$	$-45.8\times 10^{-3}$
$l_{\mathrm{Rx}}^{2}$	$6.8\times 10^{-3}$	$-103.7\times 10^{-3}$
$n_{w}^{2}$	$-109.7\times 10^{-3}$	$125.4\times 10^{-3}$
$n_{l}^{2}$	$-110.8\times 10^{-3}$	$127.2\times 10^{-3}$
$h_{a}^{2}$	$-26.5\times 10^{-3}$	$-1.2\times 10^{-3}$
$p^{2}$	$-16.0\times 10^{-3}$	$-75.1\times 10^{-3}$

**Table A3:** 32 randomly generated parameter levels inside the boundaries of Table 1. Those data are not used for calculating the regression models and are therefore denoted as test data.

No.	Parameters						Test Data	
	$r_{\mathrm{Rx}}/\mathrm{mm}$	$l_{\mathrm{Rx}}/\mathrm{mm}$	$n_{w}$	$n_{l}$	$h_{a}/\mathrm{mm}$	p	$\log(L_{\mathrm{Tx}}/\mu H)$	$\log(U_{\mathrm{Rx}}/\mathrm{mV})$
1	18.15	6.38	4	3	1.02	7	2.513	2.539
2	19.06	5.23	4	3	2.10	4	2.537	1.936
3	11.27	5.49	3	2	1.36	3	0.797	2.889
4	19.13	9.12	2	3	2.32	4	1.443	2.716
5	16.32	8.47	2	2	0.86	2	0.446	3.781
6	10.98	6.59	3	4	1.03	2	2.071	2.580
7	12.78	9.75	4	3	0.79	9	2.379	2.536
8	15.47	5.17	3	3	0.77	9	1.786	2.750
9	19.58	7.19	4	2	2.24	6	1.912	2.307
10	19.65	6.91	2	3	1.66	1	1.315	2.958
11	11.58	8.83	4	3	1.60	3	2.256	2.373
12	19.71	8.98	3	3	0.79	4	2.114	3.142
13	19.57	5.93	2	3	2.21	8	1.362	2.200
14	14.85	7.45	3	3	1.74	1	1.838	2.623
15	18.00	7.23	3	3	1.20	1	1.943	2.885
16	11.42	8.23	3	2	1.53	2	0.934	3.051
17	14.22	8.55	3	2	1.30	6	1.154	2.998
18	19.16	8.77	4	4	0.65	7	3.183	2.605
19	17.92	6.38	3	2	0.98	6	1.222	3.219
20	19.59	8.40	3	4	0.75	5	2.644	2.847
21	16.56	8.28	4	3	0.87	5	2.491	2.765
22	10.36	5.81	3	4	0.98	3	1.999	2.499
23	18.49	5.59	4	2	1.33	7	1.754	2.651
24	19.34	7.49	4	3	0.60	3	2.513	2.958
25	16.79	9.80	3	2	2.31	6	1.370	2.529
26	17.58	6.70	3	4	2.39	2	2.542	1.991
27	17.43	7.93	2	2	1.48	4	0.559	3.393
28	13.92	6.12	2	4	1.48	6	1.572	2.417
29	16.55	8.76	3	4	1.18	7	2.572	2.457
30	11.71	6.28	4	4	2.30	2	2.709	1.655
31	17.06	7.53	4	2	1.24	8	1.781	2.730
32	10.32	8.50	2	3	0.72	7	0.881	3.178

## Appendix B. D-Optimal Design for the Angular Error

Analogously to Appendix A, Table A4 shows the full design matrix in normalized notation and the response values used for the calculation of the regression models of the 0th and 4th harmonic amplitude of the angular error. The regression coefficients for the full models are listed in Table A5 and the test data can be obtained from Table A6.

**Table A4:** D-optimal design with 25 experiments. The parameter levels are normalized from −1 to +1. In the experiments those levels correspond to the values in Table 1, although $h_{a}$ is not considered in this design.

No.	Design Matrix																					Training Data	
	I	$r_{\mathrm{Rx}}$	$l_{\mathrm{Rx}}$	$n_{w}$	$n_{l}$	p	$r_{\mathrm{Rx}}l_{\mathrm{Rx}}$	$r_{\mathrm{Rx}}n_{w}$	$r_{\mathrm{Rx}}n_{l}$	$r_{\mathrm{Rx}}p$	$l_{\mathrm{Rx}}n_{w}$	$l_{\mathrm{Rx}}n_{l}$	$l_{\mathrm{Rx}}p$	$n_{w}n_{l}$	$n_{w}p$	$n_{l}p$	$r_{\mathrm{Rx}}^{2}$	$l_{\mathrm{Rx}}^{2}$	$n_{w}^{2}$	$n_{l}^{2}$	$p^{2}$	$H_{0}/\deg$	$\log(H_{4}/\deg)$
1	+1	−1	+1	−1	+1	+1	−1	+1	−1	−1	−1	+1	+1	−1	−1	+1	+1	+1	+1	+1	+1	−1.110	−0.541
2	+1	+1	−1	+1	−1	+1	−1	+1	−1	+1	−1	+1	−1	−1	+1	−1	+1	+1	+1	+1	+1	−0.519	−2.018
3	+1	−1	−1	+1	−1	−1	+1	−1	+1	+1	−1	+1	+1	−1	−1	+1	+1	+1	+1	+1	+1	0.005	−1.764
4	+1	+1	+1	−1	−1	+1	+1	−1	−1	+1	−1	−1	+1	+1	−1	−1	+1	+1	+1	+1	+1	−0.349	−0.659
5	+1	−1	−1	−1	−1	+1	+1	+1	+1	−1	+1	+1	−1	+1	−1	−1	+1	+1	+1	+1	+1	−0.849	−1.607
6	+1	+1	+1	+1	+1	+1	+1	+1	+1	+1	+1	+1	+1	+1	+1	+1	+1	+1	+1	+1	+1	−0.316	−0.958
7	+1	+1	+1	+1	−1	−1	+1	+1	−1	−1	+1	−1	−1	−1	−1	+1	+1	+1	+1	+1	+1	0.083	−0.296
8	+1	+1	−1	+1	+1	−1	−1	+1	+1	−1	−1	−1	+1	+1	−1	−1	+1	+1	+1	+1	+1	0.303	−2.387
9	+1	0	+1	+1	−1	+1	0	0	0	0	+1	−1	+1	−1	+1	−1	0	+1	+1	+1	+1	−0.647	−0.692
10	+1	+1	+1	−1	+1	−1	+1	−1	+1	−1	−1	+1	−1	−1	+1	−1	+1	+1	+1	+1	+1	0.288	−0.529
11	+1	+1	−1	−1	−1	−1	−1	−1	−1	−1	+1	+1	+1	+1	+1	+1	+1	+1	+1	+1	+1	0.457	−1.915
12	+1	−1	+1	−1	−1	−1	−1	+1	+1	+1	−1	−1	−1	+1	+1	+1	+1	+1	+1	+1	+1	0.129	−0.323
13	+1	−1	−1	+1	+1	+1	+1	−1	−1	−1	−1	−1	−1	+1	+1	+1	+1	+1	+1	+1	+1	−0.807	−1.940
14	+1	−1	0	0	+1	−1	0	0	−1	+1	0	0	0	0	0	−1	+1	0	0	+1	+1	0.178	−1.120
15	+1	+1	+1	0	+1	0	+1	0	+1	0	0	+1	0	0	0	0	+1	+1	0	+1	0	−0.108	−0.832
16	+1	−1	+1	+1	+1	−1	−1	−1	−1	+1	+1	+1	−1	+1	−1	−1	+1	+1	+1	+1	+1	0.071	−0.615
17	+1	−1	−1	−1	0	−1	+1	+1	0	+1	+1	0	+1	0	+1	0	+1	+1	+1	0	+1	0.390	−2.359
18	+1	−1	+1	+1	0	0	−1	−1	0	0	+1	0	0	0	0	0	+1	+1	+1	0	0	−0.484	−0.620
19	+1	−1	−1	−1	+1	0	+1	+1	−1	0	+1	−1	0	−1	0	0	+1	+1	+1	+1	0	−0.223	−2.419
20	+1	0	0	−1	−1	0	0	0	0	0	0	0	0	+1	0	0	0	0	+1	+1	0	−0.207	−1.002
21	+1	0	−1	0	0	+1	0	0	0	0	0	0	−1	0	0	0	0	+1	0	0	+1	−0.473	−2.135
22	+1	−1	0	+1	−1	+1	0	−1	+1	−1	0	0	0	−1	+1	−1	+1	0	+1	+1	+1	−1.147	−0.916
23	+1	+1	−1	−1	+1	+1	−1	−1	+1	+1	+1	−1	−1	−1	−1	+1	+1	+1	+1	+1	+1	−0.158	−2.786
24	+1	−1	+1	0	−1	+1	−1	0	+1	−1	0	−1	+1	0	0	−1	+1	+1	0	+1	+1	−1.370	−0.407
25	+1	+1	0	+1	0	−1	0	+1	0	−1	0	0	0	0	−1	0	+1	0	+1	0	+1	0.173	−0.887

**Table A5:** Regression coefficients for the model of the 0th and 4th harmonic amplitude. The coefficients are given in normalized notation. Hence, the parameter levels must be normalized from −1 to +1, according to the boundaries listed in Table 1. Note that the values for the model $\tilde{H_{4}}$ were calculated for logarithmized data (exponential model).

Term	Regression Coefficient $\beta_{i}$	
	$\tilde{H_{0}}$	$\tilde{H_{4}}$
*I*	$-201.5\times 10^{-3}$	1.156
$r_{\mathrm{Rx}}$	$198.7\times 10^{-3}$	$-96.9\times 10^{-3}$
$l_{\mathrm{Rx}}$	$-74.2\times 10^{-3}$	$788.1\times 10^{-3}$
$n_{w}$	$-61.3\times 10^{-3}$	$30.9\times 10^{-3}$
$n_{l}$	$69.7\times 10^{-3}$	$-191.4\times 10^{-3}$
*p*	$-442.8\times 10^{-3}$	$-49.7\times 10^{-3}$
$r_{\mathrm{Rx}}l_{\mathrm{Rx}}$	$34.8\times 10^{-3}$	$43.7\times 10^{-3}$
$r_{\mathrm{Rx}}n_{w}$	$-18.4\times 10^{-3}$	$11.0\times 10^{-3}$
$r_{\mathrm{Rx}}n_{l}$	$-8.5\times 10^{-3}$	$-37.7\times 10^{-3}$
$r_{\mathrm{Rx}}p$	$139.5\times 10^{-3}$	$118.9\times 10^{-3}$
$l_{\mathrm{Rx}}n_{w}$	$39.2\times 10^{-3}$	$-70.7\times 10^{-3}$
$l_{\mathrm{Rx}}n_{l}$	$-19.1\times 10^{-3}$	$90.5\times 10^{-3}$
$l_{\mathrm{Rx}}p$	$-7.2\times 10^{-3}$	$-78.2\times 10^{-3}$
$n_{w}n_{l}$	$42.5\times 10^{-3}$	$42.0\times 10^{-3}$
$n_{w}p$	$32.7\times 10^{-3}$	$-23.8\times 10^{-3}$
$n_{l}p$	$4.5\times 10^{-3}$	$26.3\times 10^{-3}$
$r_{\mathrm{Rx}}^{2}$	$-127.9\times 10^{-3}$	$28.7\times 10^{-3}$
$l_{\mathrm{Rx}}^{2}$	$33.7\times 10^{-3}$	$-276.3\times 10^{-3}$
$n_{w}^{2}$	$81.1\times 10^{-3}$	$-47.1\times 10^{-3}$
$n_{l}^{2}$	$-52.2\times 10^{-3}$	$23.6\times 10^{-3}$
$p^{2}$	$45.1\times 10^{-3}$	$83.4\times 10^{-3}$

**Table A6:** 25 randomly generated parameter levels inside the boundaries of Table 1, but without considering the air gap $h_{a}$. Those data are not used for calculating the regression models and are therefore denoted as test data.

No.	Parameters					Test Data	
	$r_{\mathrm{Rx}}/\mathrm{mm}$	$l_{\mathrm{Rx}}/\mathrm{mm}$	$n_{w}$	$n_{l}$	p	$H_{0}/\deg$	$\log(H_{4}/\deg)$
1	18.15	8.79	3	3	2	0.041	−0.703
2	19.06	8.72	3	2	7	−0.303	−0.815
3	11.27	6.96	3	3	3	−0.157	−1.290
4	19.13	8.28	2	3	5	−0.078	−1.006
5	16.32	5.86	2	3	2	0.208	−1.743
6	10.98	8.53	3	3	6	−0.462	−0.872
7	12.78	5.16	4	4	3	−0.207	−2.259
8	15.47	6.38	3	3	6	−0.276	−1.581
9	19.58	5.23	3	3	7	−0.278	−2.045
10	19.65	5.49	2	4	7	−0.085	−2.266
11	11.58	9.12	4	3	5	−0.366	−0.858
12	19.71	8.47	3	4	2	0.101	−0.881
13	19.57	6.59	3	4	3	0.031	−1.546
14	14.85	9.75	3	3	8	−0.452	−0.764
15	18.00	5.17	4	3	2	−0.005	−1.883
16	11.42	7.19	4	2	8	−0.761	−1.059
17	14.22	6.91	3	2	5	−0.315	−1.141
18	19.16	8.83	2	3	9	−0.280	−0.991
19	17.92	8.98	2	4	2	0.154	−0.832
20	19.59	5.93	3	4	5	−0.107	−1.935
21	16.56	7.45	4	2	2	−0.085	−0.867
22	10.36	7.23	3	3	9	−0.905	−1.074
23	18.49	8.23	4	3	1	0.148	−0.717
24	19.34	8.55	2	2	7	−0.234	−0.813
25	16.79	8.77	4	3	8	−0.394	−0.972
